# Resected Early-Onset Pancreatic Cancer: Practices and Outcomes in an International Dual-Center Study

**DOI:** 10.1245/s10434-022-12901-6

**Published:** 2022-12-07

**Authors:** Carl-Stephan Leonhardt, Benedict Kinny-Köster, Thomas Hank, Joseph R. Habib, Sami Shoucair, Ulla Klaiber, John L. Cameron, Thilo Hackert, Christopher L. Wolfgang, Markus W. Büchler, Jin He, Oliver Strobel

**Affiliations:** 1 Department of General, Visceral and Transplantation Surgery, Heidelberg University Hospital, Heidelberg, Germany; 2 Department of General Surgery, Division of Visceral Surgery, Medical University of Vienna, Vienna, Austria; 3 Department of Surgery, Johns Hopkins University School of Medicine, Baltimore, USA; 4 Department of Surgery, New York University Grossman School of Medicine and NYU Langone Health, New York, USA

## Abstract

**Background:**

Early-onset pancreatic cancer (EOPC), defined as age ≤ 45 years at diagnosis, accounts for 3% of all pancreatic cancer cases. Although differences in tumor biology have been suggested, available data are sparse and specific treatment recommendations are lacking. This study explores the clinicopathological features and oncologic outcomes of resected EOPC.

**Patients and Methods:**

Patients with EOPC undergoing resection between 2002 and 2018 were identified from the Heidelberg University Hospital and Johns Hopkins University registries. Median overall survival (OS) and recurrence-free survival (RFS) were analyzed, and prognostic factors were identified.

**Results:**

The final cohort included 164 patients, most of whom had pancreatic ductal adenocarcinoma (PDAC, *n *= 136; 82.9%) or IPMN-associated pancreatic cancer (*n *= 17; 10.4%). Twenty (12.1%) patients presented with stage 1 disease, 42 (25.6%) with stage 2, 75 (45.7%) with stage 3, and 22 (13.4%) with oligometastatic stage 4 disease. Most patients underwent upfront resection (*n *= 113, 68.9%), whereas 51 (31.1%) individuals received preoperative treatment. Median OS and RFS were 26.0 and 12.4 months, respectively. Stage-specific median survival was 70.6, 41.8, 23.8, and 16.9 months for stage 1, 2, 3, and 4 tumors, respectively. Factors independently associated with shorter OS and RFS were R1 resections and AJCC stages 3 and 4. Notably, AJCC 3-N2 and AJCC 3-T4 tumors had a median OS of 20 months versus 29.5 months, respectively.

**Conclusion:**

Despite frequently presenting with advanced disease, oncologic outcomes in EOPC patients are satisfactory even in locally advanced cancers, justifying aggressive surgical approaches. Further research is needed to tailor current guidelines to this rare population.

**Supplementary Information:**

The online version contains supplementary material available at 10.1245/s10434-022-12901-6.

Pancreatic ductal adenocarcinoma (PDAC) is estimated to become the second leading cause of cancer-related death by 2030.^1^ The average age of diagnosis peaks in the seventh decade of life, but approximately 3% of PDAC cases are diagnosed below the age of 45 years.^2^ This group, referred to as early-onset pancreatic cancer (EOPC), stands out as it is responsible for approximately 25% of all potential years of life lost owing to pancreatic cancer.^3^ In the past two decades, the age-specific incidence of EOPC has increased, with a more pronounced increase in younger age groups.^4^

Cancers affecting young adults define a distinct patient population in tumor biology as well as clinical outcomes in many tumor entities.^5–7^ For example, breast cancer diagnosed in women prior to the age of 45 years is associated with a worse prognosis independent of stage as well as histological subtype compared with elderly patients.^8^ Similarly, a diagnosis of colorectal cancer below the age of 40 years is associated with worse outcomes in stage 1, 2, and 3 tumors.^8^

Little is known about clinical outcomes of resected EOPC. National registry studies lack sufficient granularity precluding adequate differentiation between different subtypes of pancreatic cancer as well as pre-and postoperative treatment strategies.^3^ At the same time, owing to its rarity, single-center studies lack sufficient power to provide conclusive evidence. Hence, results remain conflicting. Additionally, a frequent delay in diagnosis of EOPC results in more advanced tumors.^9^ Therefore, oncological outcomes of patients with EOPC with locally advanced PDAC (LAPC) treated with an aggressive multimodal therapy including resection remain unknown.

This study aimed to investigate clinicopathologic characteristics, treatment practices, and define oncological outcomes of patients with EOPC treated with modern multimodal therapy in two high-volume centers in the USA and Germany.

## Methods

### Study Design and Study Population

This retrospective dual-center study was approved by the institutional ethics committees of Heidelberg University Hospital, Germany, as well as of the Johns Hopkins University, USA (approval numbers S-083/2021 and IRB00292313) and meets the guidelines of the responsible governmental authorities in both countries. The study was conducted according to the STROBE recommendations for observational studies.^10^ Patients aged ≤ 45 years at time of diagnosis undergoing resection for pancreatic cancer between January 2002 and December 2018 were identified from the prospectively maintained institutional registries at both institutions.

Inclusion criteria were histologically confirmed PDAC, PDAC arising from IPMN, ITPN, or MCN, adenosquamous pancreatic cancer, and other malignant pancreatic neoplasms. Furthermore, R2 resections and patients without information on preoperative treatment were excluded.

Clinicopathological features including patient’s demographics, subtype of pancreatic cancer, tumor localization, type of resection, grading, TNM classification, and AJCC/IUCC staging were extracted from the electronic medical record. Data on race and ethnicity are not routinely collected in Germany’s healthcare system and is thus not available for the HUH cohort.

### Pathologic Workup

Standardized pathology protocols exist in both institutions and were followed for processing of surgical specimen.^11,12^ Tumors were classified according to the 8th edition of the TNM staging system.^13^ The 8th editions of the AJCC/UICC cancer staging manual was used for prognostic staging, based on the pathologic staging data except M stage.^14^ Surgical margins were reported according to the revised definition of the R status.^15^

### Surgical and Oncological Management

Resectability was defined according to the recommendations of the International Study Group of Pancreatic Surgery (ISGPS).^16^ Whenever patients were deemed fit for surgery and tumors were classified as resectable on the basis of cross-sectional imaging, primary resection was attempted. Venous resection was performed in cases of suspected portal vein or superior mesenteric vein infiltration. In cases of local unresectability at time of diagnosis, induction treatment was initiated and conversion surgery attempted. Additionally, preoperative treatment was initiated on the basis of clinical trials as well as institutional preferences. Institutional practices determined choice of preoperative treatment strategy. Routinely, adjuvant chemotherapy or adjuvant chemoradiotherapy was recommended to all upfront-resected patients, the standard being a gemcitabine-based or 5-FU-based chemotherapy regimen.

### Follow-Up and Recurrence

Data regarding survival were collected by contacting national and regional tumor registries, general practitioners, and online obituaries, as well as through consultations in the specialized institutional pancreas outpatient clinics. Follow-up was last updated as of December 2020 for the Heidelberg University Hospital (HUH) cohort and May 2021 for the Johns Hopkins University (JHU) cohort.

Cross-sectional imaging was performed every 3–6 months for the first 2 years after surgery. Subsequently, cross-sectional imaging was performed annually until 5 years after surgery.

Recurrence was defined as either radiological or histological evidence of recurrent disease. First site of recurrence was documented. Recurrence was classified as local recurrence or distant metastasis (liver, lung, peritoneum, multiple distant). None of the patients had isolated distant recurrence at a location other than the liver, lung, or peritoneum. Additionally, cases of local recurrence and distant metastasis detected simultaneously formed a separate category.

Overall survival was defined as the time from the start of preoperative treatment or upfront surgery to either death from any cause or last follow-up. Recurrence-free survival was defined as time of preoperative treatment start or upfront surgery to first evidence of recurrence based on cross-sectional imaging or histology, death from any cause without evidence of recurrence, or last follow-up. Patients alive at last follow-up were censored at time of last follow-up.

### Statistical Analysis

Quantitative parameters are expressed as median and interquartile range (IQR), unless otherwise indicated. Categorical parameters are presented as absolute numbers as well as relative frequencies. Comparisons between categorical variables were performed using the Pearson chi-squared test or Fisher’s exact test, when appropriate. A univariable Cox proportional hazard regression model was used to identify predictors of median OS and recurrence. Significant categorical predictors from the univariable model were included in multivariable modeling to test independence of the predictors. AJCC stage was utilized as a composite variable derived from T stage, N stage, and M stage instead of the individual stage criteria. To determine the risk-adjusted model, conditional backwards selection (stepwise elimination of variables from the model based on the lowest *p*-value, if *p *> 0.10) was performed. Survival and recurrence curves were created using the Kaplan–Meier method, and differences (including pairwise comparisons between recurrence locations) were assessed using the log-rank test.^17^ Statistical significance was set at *p *< 0.05. IBM SPSS statistics version 25 (Armonk, NY, USA) was used for statistical analysis.

## Results

### Patient Characteristics

**Fig. 1:**
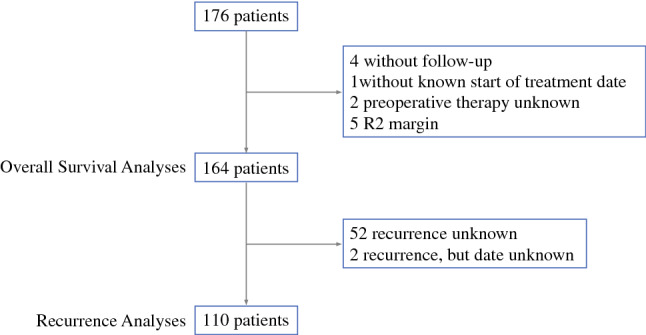
Study flowchart

Between January 2002 and December 2018, 176 patients aged ≤ 45 years underwent resection for pancreatic cancer. Five patients were excluded due to R2 resection, four due to loss to follow-up, two patients due to unknown preoperative treatment, and one patient due to unknown date of preoperative treatment start (Fig. 1).

The final study population consisted of 164 patients, 107 (65.2%) of whom were operated at HUH, while 57 (34.8%) patients were operated at JHU (Table 1). Most patients (*n *= 136, 82.9%) had classical PDAC, 17 (10.4%) PDAC arising from cystic tumors (16 IPMN-associated PDAC, 1 MCN), 8 (4.9%) adenosquamous pancreatic cancer, and 3 (1.8%) had rare subtypes of PDAC. A total of 113 (68.9%) patients underwent pancreatoduodenectomy (PD), 33 (20.1%) distal pancreatectomy (DP), and 18 (11.0%) total pancreatectomy. Venous resections were performed in 38 (23.2%) patients, arterial resections in 4 (2.4%), and combined venous and arterial resections in 7 (4.3%) patients. Postoperative 90 day mortality rate was 1.8% (*n *= 3).

**Table 1:** Clinicopathologic characteristics of the study cohort

*N*	164
Median cumulative overall survival	26.0 months (95% CI 20.0–32.0)
Postoperative 3-month mortality	3 (1.8%)
*Age at start of treatment (years)*	
Median (IQR)	41.1 (37.7–43.4)
Mean (SD)	39.9 (± 4.6)
Range	15.9–45.0
*Sex*	
Female	79 (48.2%)
Male	85 (51.8%)
*Cohort*	
HUH	107 (65.2%)
JHU	57 (34.8%)
*ASA*	
1	11 (6.7%)
2	97 (59.1%)
3	28 (17.1%)
Unknown	28 (17.1%)
*CA19-9*	
≤ 37 U/mL	55 (33.5%)
≤ 200 U/mL	43 (26.2%)
> 200 U/mL	44 (26.8%)
Unknown	22 (13.4%)
*CEA*	
≤ 2.5 ng/mL	90 (54.9%)
> 2.5 ng/mL	36 (22.0%)
Unknown	38 (23.2%)
Preoperative treatment	51 (31.1%)
Preoperative chemotherapy	47 (92.2%)
5-FU based	35 (74.5%)
Gemcitabine based	7 (14.9%)
Both (due to switch)	5 (10.6%)
Preoperative radiation	25 (49.0%), 6 unknown (11.8%)
Upfront surgery	113 (68.9%)
*Surgery*	
Pancreatoduodenectomy	113 (68.9%)
Distal pancreatectomy	33 (20.1%)
Total pancreatectomy	18 (11.0%)
*Vascular resection*	
Vein (PV/SMV)	38 (23.2%)
Artery	4 (2.4%)
Both	7 (4.3%)
None	115 (70.1%)
*R status*	
R0 (≥ 1 mm)	85 (51.8%)
R1 (including R0 CRM+)	79 (48.2%)
*Pathology*	
PDAC	136 (82.9%)
PDAC arising from cystic tumor (IPMN or MCN)	17 (10.4%)
Adenosquamous carcinoma	8 (4.9%)
Other	3 (1.8%)
*Grading*	
G1	2 (1.2%)
G2	79 (48.2%)
G3	51 (31.1%)
G4	2 (1.2%)
Unknown	30 (18.3%)
*AJCC 8th edition*	
Stage 1A	5 (3.0%)
Stage 1B	15 (9.1%)
Stage 2A	4 (2.4%)
Stage 2B	38 (23.2%)
Stage 3	75 (45.7%)
Stage 4	22 (13.4%)
Unknown	5 (3.0%)
*T stage (p or yp)*	
T1	20 (12.2%)
T2	80 (48.8%)
T3	35 (21.3%)
T4	21 (12.8%)
Unknown	8 (4.9%)
*N stage (p or yp)*	
N0	46 (28.0%)
N1	54 (32.9%)
N2	63 (38.4%)
Unknown	1 (0.6%)
*M stage*	
M0	142 (86.6%)
M1	22 (13.4%)
Adjuvant treatment	109 (66.5%), 29 unknown (17.7%)
Adjuvant chemotherapy	105 (96.3%)
5-FU based	28 (26.7%)
Gemcitabine based	74 (70.5%)
Olaparib	1 (1.0%)
Unknown regimen	2 (1.9%)
Adjuvant radiation	24 (22.0%)
*Systemic treatment*	
Preoperative only	14 (8.5%)
Adjuvant only	81 (49.4%)
Both	24 (14.6%)
None	16 (9.8%)
Preoperative, unknown adjuvant	9 (5.5%)
No preoperative, unknown adjuvant	20 (12.2%)
*RFS*	
Yes	116 (70.7%)
No	48 (29.3%)

*IQR* interquartile range, *SD* standard deviation, *HUH* Heidelberg University Hospital, *JHU* Johns Hopkins University, *AJCC* American Joint Committee on Cancer, *C19-9* carbohydrate-antigen 19-9, *CEA* carcinoembryonic antigen, *PV* portal vein, *SMV* superior mesenteric vein, *CRM* circumferential resection margin, *ASA* American Society of Anesthesiologists Classification, *5-FU* 5-fluorouracil, *IPMN* intraductal papillary mucinous neoplasm, *MCN* mucinous cystic neoplasm, *RFS* recurrence-free survival

**Fig. 2:**
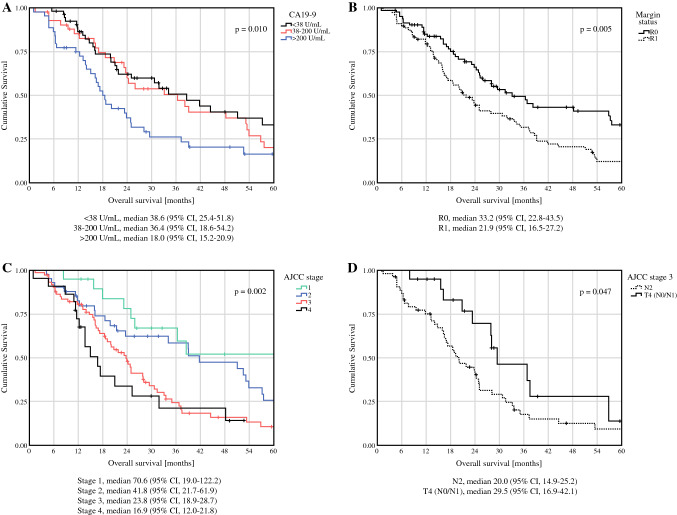
CA19-9 levels (A), margin status (B), AJCC stage (C), and AJCC stage 3 substage (D) are significantly associated with median overall survival

Median overall survival (OS) was 26.0 months from start of treatment (either surgery or preoperative treatment) and median follow-up 21.5 months (median time of preoperative treatment 5.6 months, median postoperative follow-up 19.0 months). CA19-9 levels, margin status, and AJCC stage were significantly associated with survival (*p *= 0.01, *p *= 0.005, and *p *= 0.002), respectively) (Fig. 2A, B, C). Notably, AJCC stage 3-N2 (any T) had a significantly worse median OS of 20 months than AJCC stage 3-T4 (N0 or N1) with a median OS of 29.5 months (*p *= 0.047) (Fig. 2D). No significant association was detected between CEA levels > 2.5 ng/mL and worse OS compared with ≤ 2.5 ng/mL.

### Between HUH and JHU, Survival was Similar besides Subtle Differences in Practice

**Fig. 3:**
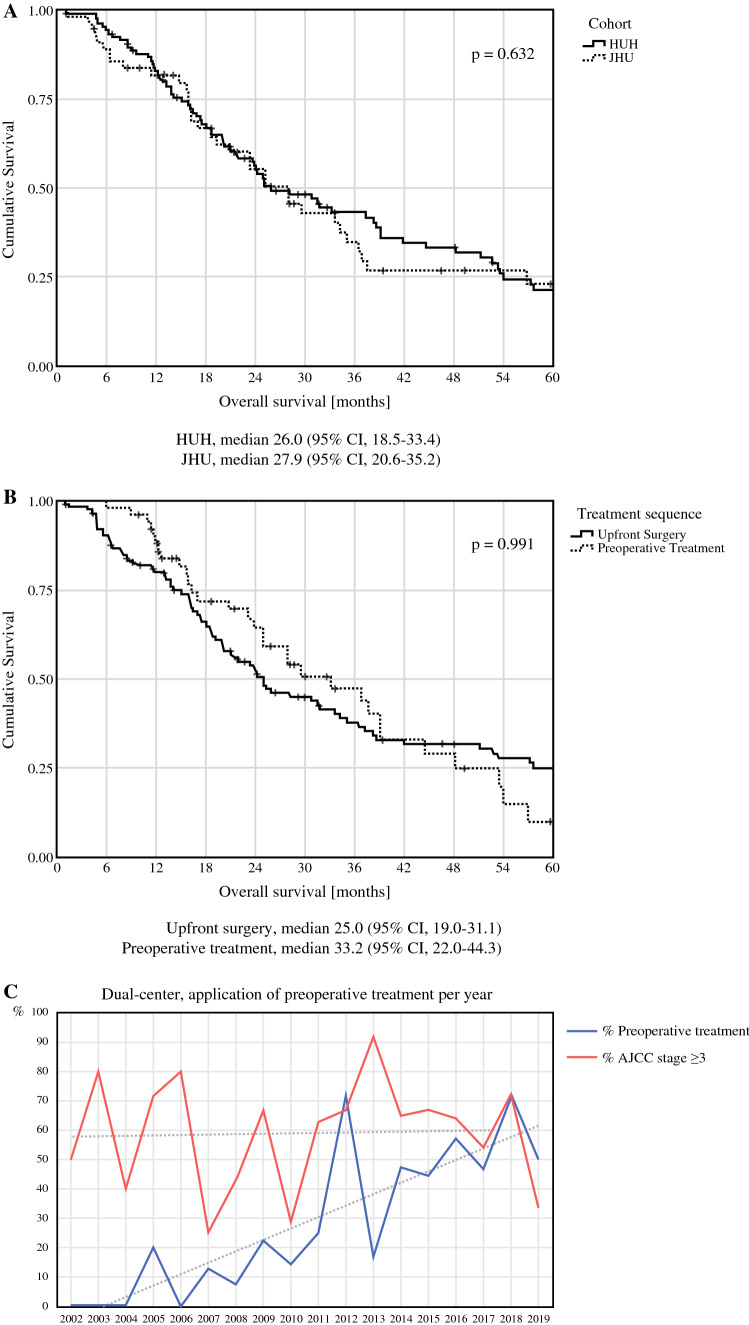
Median overall survival between the two centers (A) and between preoperatively treated and upfront resected patients (B). Over time, preoperative treatment was increasingly utilized, while resected tumor stages varied between the years with a constant distribution over time (C)

Median OS between patients treated at JHU (27.9 months) and HUH (26.0 months) did not differ (*p *= 0.63, Fig. 3A). Cumulative 5-year survival rates were 23.0 and 21.3%, respectively. Stage-specific survival did not differ significantly between the two centers, with a median OS of 34.2 months and 23.3 months for stage 2 and 3 in the JHU cohort, and 51.1 months and 23.8 months in the HUH cohort (*p *= 0.87 and *p *= 0.91, respectively). As most patients with stage 1 and 4 were treated at HUH, no comparison could be performed for these stages. Prior to resection, 51 (31.1%) patients received chemotherapy or radiotherapy. Preoperative chemotherapy regimens were either 5-FU based (*n *= 35, 74.5%) or gemcitabine based (*n *= 7, 14.9%), with some patients receiving both due to a switch in regimens (*n *= 5, 10.6%). Preoperative stereotactic body radiation therapy or chemoradiotherapy was administered to 25 (15.2%) patients. Median OS between preoperatively treated and upfront-resected patients did not differ significantly (33.2 vs. 25.0 months, *p *= 0.99, Fig. 3B). However, use of preoperative treatment has steadily increased in the last years in both centers, while no trend to treating more advanced tumors based on preoperative tumor stage was observed (Fig. 3C). Vascular resections and AJCC stage 4 tumors were significantly more common in preoperatively treated patients (*p* = 0.013 and 0.009, respectively), while adjuvant treatment was more common in the upfront surgery group (*p *= 0.015).

In total, 105 (64.0%) patients received adjuvant chemotherapy, and the majority of patients received more than three cycles of the initial chemotherapy regimen (Table 2). Adjuvant chemotherapy regimens were primarily gemcitabine based (*n *= 74, 70.5%), whereas 5-FU-based regimens were less common (*n *= 28, 26.7%). No significant differences in frequency of preoperative or adjuvant chemotherapy were detected between the two centers (*p *= 0.63). However, preoperative and adjuvant radiotherapy were significantly more common at JHU (both *p *< 0.001, respectively). In total, 16 (9.8%) patients did not receive any preoperative nor adjuvant treatment.

**Table 2:** Completion rates of adjuvant chemotherapy

*Adjuvant chemotherapy (N = 105)*	
≤ 3 cycles	19 (21.1%)
> 3 cycles	71 (78.9%)
*Unknown cycles*	*15*
*5-FU based (N = 28)*	
≤ 3 cycles	4 (18.2%)
> 3 cycles	18 (81.8%)
*Unknown cycles*	*6*
*Gemcitabine based (N = 74)*	
≤ 3 cycles	15 (22.4%)
> 3 cycles	52 (77.6%)
*Unknown cycles*	*7*

*5-FU* 5-fluorouracil

### Factors Associated with Survival

Next, predictors of reduced survival were investigated. R1 resections [hazard ratio (HR) 1.73, 95% confidence interval (CI) 1.18–2.54] and AJCC stage 3 (HR 2.74, 95% CI 1.38–5.44) and stage 4 (HR 3.57, 95% CI 1.59–8.00) tumors were significantly associated with decreased median OS (*p* = 0.005, *p* = 0.004, and *p* = 0.002, respectively). Additionally, N1 (HR 1.88, 95% CI 1.09–3.26) and N2 (HR 3.40, 95% CI 2.01–5.76) status showed significant association with decreased median OS (*p *= 0.024 and *p *= 0.0001, respectively) while PDAC arising from cystic lesions was associated with improved median OS (HR 0.37, 95% CI 0.16–0.84, *p *= 0.018). Independent predictors of survival by multivariable regression were R1 resection (*p *= 0.025) and AJCC stages 3 and 4 (*p *= 0.011 and *p *= 0.004, respectively).

### Timing and Pattern of Recurrence

**Fig. 4:**
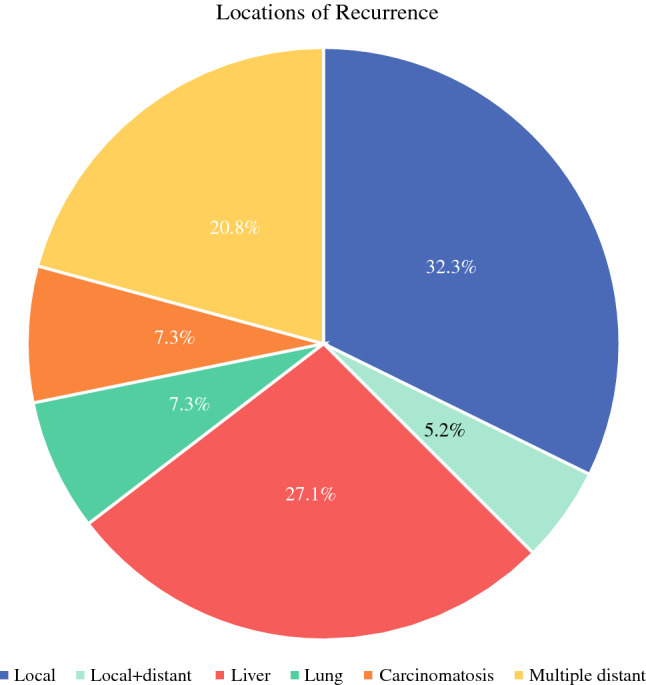
Pattern of recurrence

Median RFS was 12.4 months for the 110 patients included in the recurrence analysis (Supplementary Table 1). Recurrence occurred primarily locally (32.3%), in the liver (27.1%), as well as at multiple distant sites (20.8%), while isolated lung metastasis (7.3%) and carcinomatosis (7.3%) were rare (Fig. 4). Notably, no significant associations between recurrence location and time to recurrence were detected across the entire cohort (*p *= 0.34) as well as when performing pairwise comparisons (Supplementary Table 2). Furthermore, RFS did not differ significantly between patients who received preoperative treatment compared with those who underwent upfront surgery. Similarly, CA19-9 and CEA levels were not associated with RFS, while AJCC stage and margin status were significantly associated with RFS (Supplementary Fig. 1, *p *= 0.014 and *p *= 0.025, respectively).

### Predictors of Recurrence

Predictors of time to any first recurrence are presented in Table 3. In a multivariable regression model, R1 resection, AJCC stage 3 and stage 4 were independent predictive factors of recurrence.

**Table 3:** Prediction model time-to-event for recurrence-free survival (*N* = 110)

Margin	Univariate cox		Multivariate cox	
	HR	*p*	HR	*p*
R0 (≥ 1 mm)	1.00 (reference)		1.00 (reference)	
R1 (including R0 CRM+)	**1.58 (1.06 – 2.36)**	**0.026**	1.37 (0.80–2.35)	0.245
*Histology*				
PDAC	1.00 (reference)		1.00 (reference)	
PDAC from cystic	0.47 (0.19–1.17)	0.104	0.55 (0.20–1.53)	0.252
Adenosquamous	**3.47 (1.37–8.77)**	**0.009**	2.53 (0.92–6.93)	0.072
Other	0.29 (0.04–2.13)	0.225	0.91 (0.05–15.62)	0.950
*AJCC*				
Stage 1	1.00 (reference)		1.00 (reference)	
Stage 2	2.03 (0.88–4.68)	0.098	1.70 (0.69–4.16)	0.246
Stage 3	**2.57 (1.15–5.71)**	**0.021**	1.79 (0.73–4.37)	0.201
Stage 4	**4.15 (1.64–10.50)**	**0.003**	**3.21 (1.20–8.58)**	**0.020**
Unknown (*N* = 3)	0.78 (0.16–3.77)	0.758	0.88 (0.10–7.56)	0.907
*T stage*				
T1	1.00 (reference)		X	
T2	1.57 (0.82–2.97)	0.171		
T3	**2.93 (1.44–5.94)**	**0.003**		
T4	1.74 (0.82–3.66)	0.147		
Unknown (*N* = 3)	1.10 (0.31–3.92)	0.884		
*N stage*				
N0	1.00 (reference)		X	
N1	1.60 (0.95–2.72)	0.080		
N2	**1.96 (1.15–3.31)**	**0.013**		
*M stage*				
M0	1.00 (reference)		X	
M1	**1.98 (1.11–3.52)**	**0.020**		

*p * values below 0.05 are indicated in bold

*HR* hazard ratio, *AJCC* American Joint Committee on Cancer, *CRM* circumferential resection margin

Additional subgroup analyses for factors predicting any local or any distant recurrence were performed. None of the collected categorical variables reached statistical significance in independently predict any local recurrence (36 events, Supplementary Table 3). Univariable predictors of any distant recurrence are presented in Supplementary Table 4 (*n *= 65). R1 resection was the only independent predictor to time of any distant recurrence (HR 2.02, 95% CI 1.22–3.37, *p *= 0.007).

## Discussion

The present study details the clinical characteristics, treatment practices, and predictors of survival as well as recurrence in patients with resected EOPC using a real-life experience from two international high-volume centers of pancreatic surgery. Despite differences in therapeutic strategies, overall survival did not differ between the two centers. In both centers, an increasing trend to preoperative treatment was recognized. The median OS for patients with EOPC from the initiation of treatment was 26 months. Although no significant association was detected between location of recurrence and time to recurrence, margin status and AJCC stages 3 and 4 were independent predictors of both inferior survival and recurrence. However, even in advanced stages, a median OS of 23.8 months (AJCC stage 3) and 16.9 months (AJCC stage 4) was observed. Subgroup analysis revealed that multimodal treatment of AJCC stage 3-T4 EOPC resulted in an excellent median OS of 29.5 months with a cumulative 5 year survival rate of 14.0%.

The chosen cutoff of ≤ 45 years to define EOPC is arbitrary, yet consistent with previous reports of EOPC as well as other tumor entities.^18–20^ Considering the rarity of the condition and the lack of large-scale studies, age at diagnosis is not considered in current treatment algorithms for patients with PDAC. Moreover, national and international recommendations do not support a surgical approach for locally advanced or metastasized disease.^16,21,22^

In parallel, comorbidities as measured by the Charlson Comorbidity Index (CCI) are significantly lower in young patients, and low CCI has been associated with a higher likelihood of receiving adjuvant chemotherapy as well as lower 90 day mortality after pancreatectomy.^20,23–26^ In the present cohort, adjuvant chemotherapy was administered in 86.2% of upfront-resected patients, a rate that stands in contrast to contemporary series in which rates of adjuvant chemotherapy reach 50–70%.^27,28^ Almost 80% of patients with adjuvant-treated EOPC received more than three cycles of chemotherapy, indicative of a high completion rate of adjuvant chemotherapy and consistent with previous reports.^29^ Stage-specific median OS in the cohort ranged between 70.6 months in stage 1 EOPC and 16.9 months in AJCC stage 4 disease. Notably, resection of stage 3 EOPC resulted in an excellent median OS of 23.8 months, corresponding to LAPC or N2 disease. Subgroup analysis revealed that AJCC stage 3-T4 had a remarkable median OS of 29.5 months compared with AJCC stage-N2 disease with a median OS of 20 months. Hence, even in cases of locally advanced disease, satisfactory outcomes in patients with EOPC can be achieved in expert centers utilizing a preoperative treatment strategy combined with surgical resection. These results provide support for an aggressive multimodal therapeutic strategy in this unique patient cohort as well as underline the need to further investigate age at diagnosis for treatment recommendations of LAPC.

This study additionally highlights the treatment strategies at two tertiary referral centers in Germany and the USA. In both centers, a trend towards preoperative treatment in patients with EOPC was detected in the past decade without changes in yT and yN stages. This may be interpreted as an increasingly aggressive management of more advanced tumors by utilization of preoperative treatment, successfully leading to downstaging. Unstratified median OS between patients who underwent upfront resection and preoperatively treated patients did not differ significantly. While median OS and RFS were comparable between the two centers, preoperative and adjuvant radiotherapy were significantly more common at JHU.

Furthermore, we present detailed data on timing and patterns of recurrence in EOPC patients. In contrast to the study by Groot et al. on average-onset pancreatic cancer (AOPC), resection margin status was independently associated with distant recurrence, but not local recurrence.^30^ No association between site of recurrence and timepoint of recurrence was detected. In particular, liver metastasis did not occur earlier than lung metastasis, unlike previously reported in AOPC.^30,31^ While this finding is noteworthy, conclusions must be drawn with caution due to the relatively small sample size. Ansari et al. described a higher rate of distant metastasis in patients with EOPC than in patients with AOPC; however, no data on the location of metastasis were reported.^32^

Outcomes of patients with EOPC versus patients with AOPC remain unclear. A recent analysis of the National Cancer Database of the American College of Surgeons showed that patients with pancreatic cancer aged < 39 years had improved survival compared with those 40–50 years and > 50 years of age.^29^ Similarly, Duffy et al. reported longer survival in 35 resected patients aged ≤ 45 years with stage 1–2 pancreatic cancer relative to data from the literature.^18^ In 2013, He et al. published an earlier series assessing the outcomes in patients with resected EOPC. The investigators reported an increased median OS compared with a reference cohort of patients aged ≥ 70 years (19 vs. 16 months).^20^

Other studies could not corroborate these findings. On the contrary, some series reported reduced OS of patients with EOPC when adjusting for patient-specific factors. Using a 50 year age cutoff, Ansari et al. reported reduced OS for patients with resected EOPC compared with controls ≥ 50 years of age using propensity score matching. For patients who did not undergo resection, no significant difference in survival was detected between EOPC and later-onset pancreatic cancer.^32^ Similarly, a recent single-center analysis did not report a difference in median OS and stage-specific survival in patients < 50 years of age compared with elderly patients.^24^

Recent reports hint to a different tumor biology and genetic alterations in EOPC compared with AOPC.^33,34^ Interestingly, in a small sample of 17 patients with EOPC from a personalized oncology program, 4 patients harbored KRAS wild-type alleles. Potentially actionable oncogenic fusions were identified in all four KRAS wild-type tumors, whereas none was identified in KRAS mutated tumors.^35^ A comprehensive single-center study at MSKCC (NY, USA) comprising 450 patients with EOPC, of whom 125 underwent surgical resection, reported a median OS of 28 months for resectable disease and 18 months for unresectable locally advanced disease.^36^ Patients who underwent somatic molecular testing had no detectable KRAS mutation in 15.9% as opposed to only 5.4% for all PDAC, thus supporting the hypothesis of a distinct molecular pathogenesis of EOPC.^36^ Despite a potentially distinct tumor biology, our study confirmed that pathological prognostic factors of median OS including N1/N2, R1, and tumor stages 3 and 4 are comparable between EOPC and AOPC.

This study has several strengths: (i) It is the largest study describing outcomes in EOPC patients who underwent resection. Previous studies suffered from small sample sizes, thus hampering conclusions, or were based on regional or national registeries, hence lacking sufficient granularity. (ii) It is the first study to describe patterns of recurrence in patients with EOPC and identifies independent predictors of recurrence and survival.

However, the limitiations of this study need to be recognized. It is conceivable that younger patients will be better informed about treatment options and thus more frequently seek treatment at tertiary referral centers, resulting in a skewed patient population. Addititionally, no information on familial or hereditary pancreatic cancer was available. In particular, no details regarding molecular characteristics were available. More recent cases at JHU were tested for recommended germline mutations including BRCA1/2, in line with the most recent NCCN guideline.^21^ However, similar information was not available in the HUH cohort as genetic testing in suspected cases of familial pancreatic cancer has only recently been incorporated into the German S3 guideline.^37^ Although certain hereditary syndroms such as familial atypical mole and multiple melanoma syndrom harboring germline mutations can cause premature manifestation of pancreatic cancer, familial pancreatic cancer only seems to result in an earlier onset of 6 years compared with AOPC.^38^ Furthermore, no comprehensive assessment of risk factors for PDAC, including smoking, diabetes mellitus, alcohol consumption, and pancreatitis, was feasible owing to the dual-center study design.

## Conclusions

Oncological outcomes in patients with resected EOPC are satisfactory despite frequently presenting with advanced tumors. An excellent median OS of 70.6 months in AJCC stage 1 and 29.5 months of AJCC stage 3-T4 (N0/N1) tumors support an aggressive surgical approach in this patient population, even in LAPC. Classical prognosticators of AOPC were confirmed, and R1 resection was identified as an independent predictor of recurrence. However, questions regarding early-onset specific tumor biology remain, and additional research is necessary. It will be important to study molecular characteristics in comparison with AOPC to identify specific actionable cancer vulnerabilities in EOPC. Similarly, the contribution of familial PDAC should be tested routinely. As patients with early-onset cancer tend to be rare at single centers and underrepresented in clinical trials, age-group-specific multicenter trials are necessary.

## Supplementary Information

Below is the link to the electronic supplementary material.

Supplementary file1 (TIFF 14840 kb)

Supplementary file2 (DOCX 17 kb)

## Data Availability

Research data supporting this publication are available from the corresponding author upon request.
