# Supplementary figures and images for: Resected Early-Onset Pancreatic Cancer: Practices and Outcomes in an International Dual-Center Study
Source: Ann Surg Oncol. 2022 Dec 7;30(4):2433–43. doi: 10.1245/s10434-022-12901-6 (PMC10027827; doi:10.1245/s10434-022-12901-6)

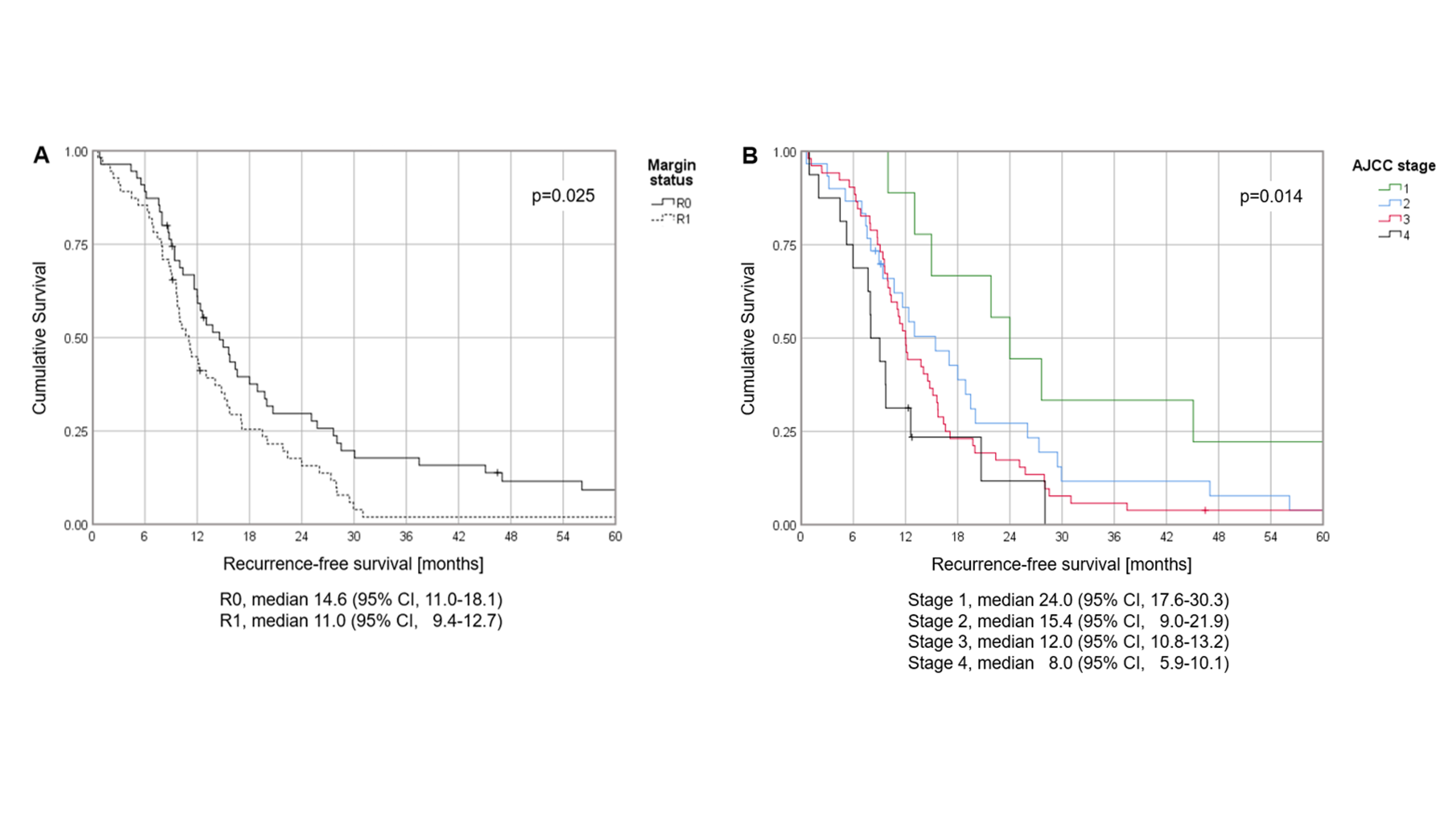

Supplement: Supplementary file 1 — Supplementary file1 (TIFF 14840 kb) [file 10434_2022_12901_MOESM1_ESM.tiff]
