# Supplementary material for: Resected Early-Onset Pancreatic Cancer: Practices and Outcomes in an International Dual-Center Study
Source: Ann Surg Oncol. 2022 Dec 7;30(4):2433–43. doi: 10.1245/s10434-022-12901-6 (PMC10027827; doi:10.1245/s10434-022-12901-6)
Supplement: Supplementary file 2 — Supplementary file2 (DOCX 17 kb) [file 10434_2022_12901_MOESM2_ESM.docx]

**Supplementary Figures**

**Suppl. Figure 1:** Resection margins **(A)** and AJCC stage **(B)** are significantly associated with recurrence-free survival.

**Suppl. Table 1:** Clinicopathologic Characteristics of Patients included in the Recurrence Analyses

| N | 110 |
| --- | --- |
| Median cumulative overall survival | 27.9 months (95% CI, 21.8 – 34.0) |
| Postop. 3-month mortality | 0 |
| Median cumulative recurrence-free survival | 12.4 (95% CI, 10.1 – 14.6) |
| Recurrence  Yes  Local (isolated)  Liver (isolated)  Lung (isolated)  Carcinomatosis (isolated)  Multiple distant (no local)  Local+distant  No | 96 (87.3%)  31 (32.3%)  26 (27.1%)  7 (7.3%)  7 (7.3%)  20 (20.8%)  5 (5.2%)  14 (12.7%) |
| Age at start of treatment [years]  Median (IQR)  Mean (SD)  Range | 41.23 (37.73 – 43.50)  39.95 (± 4.67)  15.94 – 44.85 |
| Sex  Female  Male | 53 (48.2%)  57 (51.8%) |
| Cohort  HUH  JH | 65 (59.1%)  45 (40.9%) |
| ASA  1  2  3  Unknown | 7 (6.4%)  62 (56.4%)  21 (19.1%)  20 (18.2%) |
| CA19-9  ≤37 U/mL  ≤200 U/mL  >200 U/mL  Unknown | 37 (33.6%)  27 (24.5%)  29 (26.4%)  17 (15.5%) |
| CEA  ≤2.5 ng/mL  >2.5 ng/mL  Unknown | 58 (52.7%)  23 (20.9%)  29 (26.4%) |
| Preoperative treatment  Preoperative chemotherapy  5FU-based  Gemcitabine-based  Both (due to switch)  Preoperative radiation  Upfront surgery | 38 (34.5%)  35 (92.1%)  28 (80.0%)  4 (11.4%)  3 (8.6%)  19 (50.0%), 5 unknown (13.2%)  72 (65.5%) |
| Surgery  Pancreatoduodenectomy  Distal pancreatectomy  Total pancreatectomy | 78 (70.9%)  20 (18.2%)  12 (10.9%) |
| Vascular Resection  Vein (PV/SMV)  Artery  Both  None | 26 (23.6%)  2 (1.8%)  5 (4.5%)  77 (70.0%) |
| R status  R0 (≥1 mm)  R1 (including R0 CRM+) | 55 (50.0%)  55 (50.0%) |
| Pathology  PDAC  PDAC arising from cystic tumor (IPMN or MCN)  Adenosquamous carcinoma  Other | 95 (86.4%)  8 (7.3%)  5 (4.5%)  2 (1.8%) |
| Grading  G1  G2  G3  Unknown | 2 (1.8%)  54 (49.1%)  35 (31.8%)  19 (17.3%) |
| AJCC 8^th^ edition  Stage 1A  Stage 1B  Stage 2A  Stage 2B  Stage 3  Stage 4  Unknown | 4 (3.6%)  5 (4.5%)  2 (1.8%)  28 (25.5%)  52 (47.3%)  16 (14.5%)  3 (2.7%) |
| T stage  T1  T2  T3  T4  Unknown | 15 (13.6%)  28 (43.6%)  27 (24.5%)  17 (15.5%)  3 (2.7%) |
| N stage  N0  N1  N2 | 27 (24.5%)  42 (38.2%)  41 (37.3%) |
| M stage  M0  M1 | 94 (85.5%)  16 (14.5%) |
| Adjuvant treatment  Adjuvant chemotherapy  5FU-based  Gemcitabine-based  Olaparib  Unknown regimen  Adjuvant radiation | 90 (81.8%), 2 unknown (1.8%)  86 (95.6%)  21 (24.4%)  62 (72.1%)  1 (1.2%)  2 (2.3%)  20 (22.2%) |
| Systemic treatment  Preoperative-only  Adjuvant-only  Both  None  Preoperative, unknown adjuvant  No preoperative, unknown adjuvant | 12 (10.9%)  63 (57.3%)  23 (20.9%)  10 (9.1%)  0 (0%)  2 (1.8%) |

**Legend:** IQR: Interquartile Range; SD: Standard Deviation; HUH: Heidelberg University Hospital; JHU: Johns Hopkins University; AJCC: American Joint Committee on Cancer; CA19-9: Carbohydrate-Antigen 19-9; CEA: Carcinoembryonic antigen; PV: Portal Vein; SMV: Superior Mesenteric Vein; CRM: Circumferential Resection Margin; ASA: American Society of Anesthesiologists Classification; 5-FU: 5-Fluorouracil; IPMN: Intraductal Papillary Mucinous Neoplasm; MCN: Mucinous Cystic Neoplasm; RFS: Recurrence-free Survival.

**Supplementary Table 2:** Pairwise comparisons between timing and location of recurrence (n=96) and associated p-values.

|  | Local-only | Liver-only | Lung-only | Carcinomatosis-only | Multiple distant | Local+distant |
| --- | --- | --- | --- | --- | --- | --- |
| Local-only | X | X | X | X | X | X |
| Liver-only | 0.676 | X | X | X | X | X |
| Lung-only | 0.290 | 0.557 | X | X | X | X |
| Carcinomatosis-only | 0.824 | 0.469 | 0.216 | X | X | X |
| Multiple distant | 0.059 | 0.175 | 0.132 | 0.775 | X | X |
| Local+distant | 0.271 | 0.795 | 0.732 | 0.409 | 0.232 | X |

**Supplementary Table 3:** Prediction model time-to-any local recurrence (N=110, local only and local+distant = 36 events)

|  | Univariate Cox | | Multivariate Cox | |
| --- | --- | --- | --- | --- |
|  | HR | p | HR | p |
| CA19-9 continuous per U/mL (N=93) | **1.00 (1.00 – 1.00)** | **0.044** | X |  |
| CEA per ng/mL (N=81) | **1.06 (1.02 – 1.11)** | **0.006** | X |  |

**Legend:** HR: Hazard Ratio; CA19-9: Carbohydrate-Antigen 19-9; CEA: Carcinoembryonic antigen

**Supplementary Table 4:** Prediction model time-to-any distant recurrence (N=110, liver only, lung only, carcinomatosis only, multiple distant and local+distant = 65 events)

|  | Univariate Cox | | Multivariate Cox | |
| --- | --- | --- | --- | --- |
|  | HR | p | HR | p |
| Margin  R0 (≥1 mm)  R1 (including R0 CRM+) | 1.00 (reference)  **2.06 (1.24 – 3.42)** | **0.005** | 1.00 (reference)  **2.04 (1.05 – 3.97)** | **0.036** |
| Histology  PDAC  PDAC from cystic  Adenosquamous  Other | 1.00 (reference)  0.59 (0.21 – 1.66)  **4.79 (1.86 – 12.32)**  0.44 (0.06 – 3.25) | 0.318  **0.001**  0.421 | 1.00 (reference)  0.69 (0.21 – 2.27)  2.49 (0.69 – 9.01)  0.58 (0.04 – 9.47) | 0.536  0.164  0.700 |
| AJCC  Stage 1  Stage 2  Stage 3  Stage 4  Unknown (N=3) | 1.00 (reference)  2.33 (0.78 – 6.95)  2.77 (0.96 – 7.95)  **5.42 (1.68 – 17.50)**  1.32 (0.24 – 7.23) | 0.130  0.059  **0.005**  0.749 | 1.00 (reference)  1.50 (0.45 – 5.05)  1.94 (0.60 – 6.25)  3.55 (0.99 – 12.71)  2.21 (0.22 – 22.12) | 0.512  0.270  0.051  0.501 |
| T stage  T1  T2  T3  T4  Unknown (N=3) | 1.00 (reference)  1.65 (0.76 – 3.60)  **2.56 (1.08 – 6.07)**  1.05 (0.38 – 2.93)  1.69 (0.44 – 6.43) | 0.207  **0.033**  0.921  0.443 | X |  |
| N stage  N0  N1  N2 | 1.00 (reference)  1.82 (0.93 – 3.56)  **2.25 (1.15 – 4.38)** | 0.083  **0.018** | X |  |
| M stage  M0  M1 | 1.00 (reference)  **2.33 (1.20 – 4.51)** | **0.013** | X |  |

**Legend:** HR: Hazard Ratio; AJCC: American Joint Committee on Cancer; CRM: Circumferential Resection Margin; ASA: American Society of Anesthesiologists Classification; 5-FU: 5-Fluorouracil; IPMN: Intraductal Papillary Mucinous Neoplasm; MCN: Mucinous Cystic Neoplasm; RFS: Recurrence-free Survival
